# Acellular fraction of ovarian cancer ascites induce apoptosis by activating JNK and inducing BRCA1, Fas and FasL expression in ovarian cancer cells

**DOI:** 10.18632/oncoscience.31

**Published:** 2014-04-30

**Authors:** Marie Cohen, Sandra Pierredon, Christine Wuillemin, Florence Delie, Patrick Petignat

**Affiliations:** ^1^ Department of Gynecology-Obstetrics, faculty of medicine, Geneva, Switzerland; ^2^ School of Pharmaceutical Sciences, University of Geneva, University of Lausanne, Geneva,Switzerland.

**Keywords:** apoptosis, ascites, JNK, Fas, FasL, BRCA1

## Abstract

Acellular fraction of ascites might play an active role in tumor development. Nevertheless the mechanisms involved in the tumor-modulating properties are still controversial. Here, we demonstrate that malignant ascites from 8 patients with epithelial ovarian cancer did not influence proliferative or invasive properties of ovarian cancer cells, but promoted H_2_O_2_-induced apoptosis and increased sensitivity to paclitaxel. Malignant ascites induced BRCA1, Fas and FasL expression and phosphorylation of JNK, but not the activation of caspase pathway. Ascites-induced apoptosis of ovarian cancer cells was strongly inhibited by a JNK inhibitor suggesting a critical role of JNK pathway in ascite-induced apoptosis. The use of siRNA JNK confirmed the importance of JNK in ascites-induced Fas and FasL expression. These results demonstrate that malignant ascites induce apoptosis of ovarian cancer cells and encourage us to think about the clinical management of ovarian cancer patients with malignant ascites.

## INTRODUCTION

Ovarian cancer is the fifth leading cause of cancer-related death in the world and has the highest mortality of any of the gynecologic cancers. The most common form of ovarian cancer is epithelial ovarian cancer (EOC). Current treatments of advanced ovarian carcinoma are surgery and chemotherapy. Initially, more than 70% of treated patient respond to treatment and the disease relapses in about 70% of the cases. However resistance to second therapy is found in most cases. Late stage of disease at diagnosis and relative resistance to secondary chemotherapy are displayed by most EOC and therefore represent a significant clinical challenge.

More than one-third of ovarian cancer patients present large amount of ascites at the time of diagnosis [1]. Ascites is an exudative fluid composed of a cellular fraction consisting of ovarian cancer cells, lymphocytes, and mesothelial cells and an acellular fraction containing angiogenic and growth factors, bioactive lipids, cytokines and extracellular matrix components. All these factors are known to individually induce cell growth, invasion and/or survival suggesting that ascites might play an active role in development and progression of EOC. This hypothesis was supported by clinical observation that ascites' presence correlates with higher tumor spread [2].

Studies conducted *in vitro* on EOC cell lines have shown that the acellular fraction of EOC ascites could affect cell proliferation, apoptosis, migration, invasion, and *de novo* drug resistance [3-7]. The first study conducted in 2007 suggests that both positive and negative regulators of tumor behavior may be present in the acellular fraction of ascites [3]. In fact, these data demonstrate contrasting effects of ascites on cell invasion, migration and proliferation [3, 7]. These data are insufficient to clarify the effective role of the acellular fraction of ascites on the behavior of cells mainly because of the variability of clinical characteristic.

It has also been shown that pretreatment of ovarian cancer cells with malignant ascites can significantly block TRAIL induced apoptosis [4-6]. This prosurvival effect seems to be mediated through PI3K/Akt-dependent pathway [4]. Paradoxically ascitic fluids from ovarian cancer revealed a net anti-angiogenic property in a chick chorioallantoic membrane (CAM) assay [8]. This effect is attributed in part to immunopurified fibrinogen products (FDPs).

Unlike other solid malignancies, where ascites portends a universally poor prognosis, patients with malignant ascites secondary to ovarian cancer at the time of diagnosis represent an exception because they have a significantly better survival, with a median of almost 2 years, than those with gastrointestinal cancer, in whom median survival was almost 3 months (*p* = 0.0001) [9]. This observation may suggest that acellular fraction of ovarian malignant ascites compared to the ascites from other solid malignancies could delay the progression of the disease.

Thus, the acellular fraction of ovarian cancer ascites could affect the tumor microenvironment but the mechanisms involved in these tumor-modulating properties are still controversial. These information are particularly interesting since the reinfusion of acellular fraction of ascites in ovarian cancer patients is a method developed and used in Japan [10].

Based on the observation that cellular fraction of EOC ascites may affect tumor microenvironment, we exposed EOC cell line SKOV3 to acellular fraction of ascites from 8 patients diagnosed with serous ovarian adenocarcinoma (grade 2 and 3) and evaluated in vitro cell proliferation, invasion and apoptosis and in vivo tumour development on chick chorioallantoid membrane (CAM).

## RESULTS

### Effects of ascites on cell behaviour

**Fig 1:**
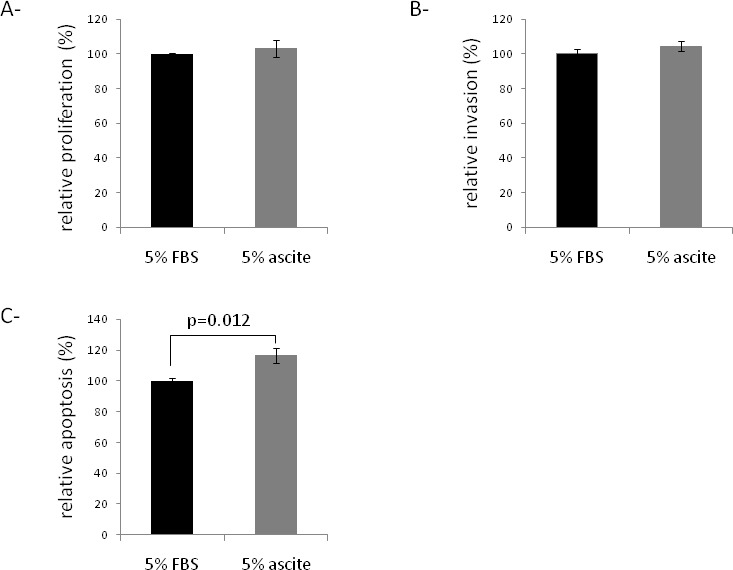
Effects of the 8 ascites tested separately on behavior of SKOV3 cells (A) Effect of ascites on the proliferation of the SKOV3 cell line. The proliferative potential of cells was evaluated after 48 hours using Wst-1 reagent and compared to 5% FBS. (B) Effect of ascites on the invasion of the SKOV3 cell line. The invasive potential of cells was determined by the ability to invade collagen membrane, normalized by proliferation assay after 48 hours, and compared to 5% FBS. (C) Effect of ascites on the early apoptosis of the SKOV3 cell line. The apoptosis of cells was determined by apopercentage assay after 48 hours, and compared to 5% FBS.

Ascites from 8 EOC patients were studied to determine their effect on SKOV3 cell proliferation (Figure 1A), invasion (Figure 1B), H_2_O_2_-induced apoptosis (Figure 1C). The influence of ascites on cell properties was expressed in percentage to medium supplemented with 5% FBS. In SKOV3 cells, no difference in proliferation and invasion was observed between cells treated with 5% ascites and control cells (Figure 1A and 1B). In contrast, ascites treatment increased H_2_O_2_-induced apoptosis (Figure 1C).

**Fig. 2:**
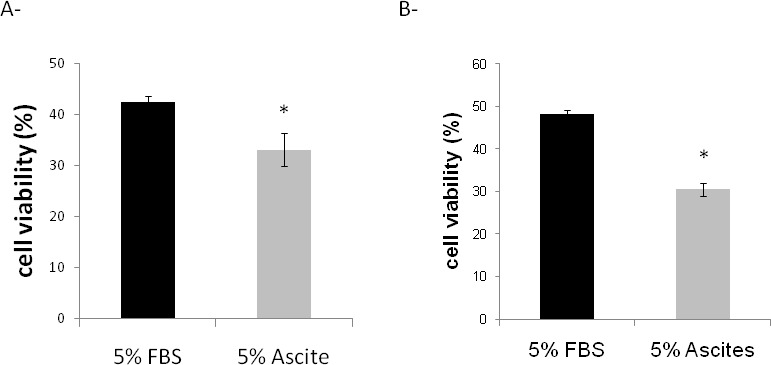
Effect of ascites on cell viability of paclitaxel-treated SKOV3 (A) and OCAM (B) cells The cell viability was determined by MTT assay after 48 hours, and compared to cells incubated with 5% FBS without paclitaxel. *p≤0.05.

Ascites were then tested on paclitaxel sensitivity of SKOV-3 (Figure 2A) and OCAM cells (Figure 2B). In both cells, malignant ascites significantly increased the cellular sensitivity to paclitaxel (Figure 2). These observations suggest that malignant ascites could induce apoptosis of ovarian cancer cells.

### Effects of EOC ascites on BRCA1, Fas and FasL expression

**Fig. 3:**
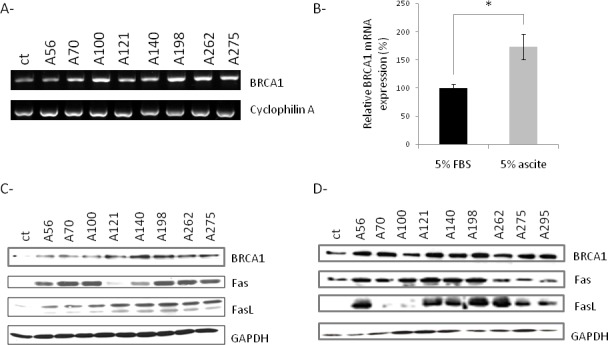
Effects of ascites on BRCA1, Fas and FasL expression (A) Evaluation of BRCA1 mRNA of SKOV3 cells treated with 5% FBS (ct) or 5% ascites by RT-PCR. (B) The intensity of the BRCA1 bands from three independent experiments was quantified and normalized to cyclophilin A. (C) Evaluation of BRCA1, Fas, FasL levels of SKOV3 cells treated with 5% FBS (ct) or 5% ascites by western blot analysis. (D). Evaluation of BRCA1, Fas, FasL levels of OCAM cells treated with 5% FBS (ct) or 5% ascites by western blot analysis. *p≤0.05.

To determine the apoptotic pathway activated by ascites, SKOV3 cells were treated or not with ascites from 8 EOC patients. PCR and western blot analysis demonstrated a significant increase in BRCA1 mRNA and protein levels in SKOV3 treated cells compared to control cells (Figure 3A, 3B, 3C). FasL/Fas interactions have previously been shown to play an important role in mediating BRCA1-dependent apoptosis in breast and ovarian cancer cells [11]. We next investigated Fas and FasL expression in SKOV3 cells treated or not with malignant ascites. We observed an increase of Fas and FasL levels in ascites-treated SKOV3 cells compared to control cells (Figure 3C). These observations were confirmed on OCAM cells (Figure 3D).

### EOC ascites-dependent apoptotic pathway is caspase 3 independent

**Fig. 4:**
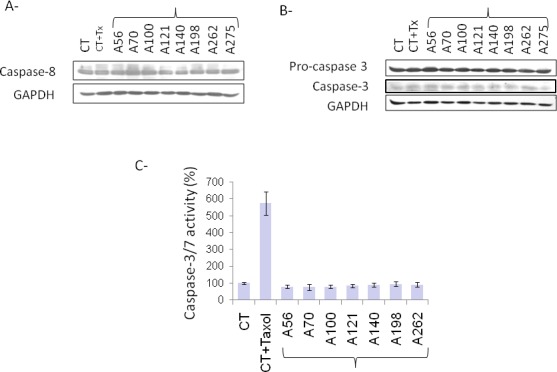
Caspase-independent ascite-induced apoptosis (A) Evaluation of caspase 8 and GAPDH levels in SKOV3 cells treated with 5% FBS (CT), 5% FBS and 100 nM Paclitaxel (CT+Taxol) or 5% ascites by western blot analysis. (B) Evaluation of procaspase 3, caspase 3 and GAPDH levels in SKOV3 cells treated with 5% FBS (CT), 5% FBS and 100 nM Paclitaxel (CT+Taxol) or 5% ascites by western blot analysis. (C) Evaluation of caspase 3 and 7 activities in SKOV3 cells treated with 5% FBS (CT), 5% FBS and 100 nM paclitaxel (CT+Taxol) or 5% ascites. Results are expressed in percentage of controls for each sample of ascites.

Two pathways of Fas-dependent caspase activation have already been described [11-13]. After caspase 8 activation, one is followed by direct activation of effector caspases and the second involves cleavage of Bcl-2 family member Bid that induced release of cytochrome c from mitochondria and subsequent activation of effector caspases. To assess the activation of caspase pathway in response to ascites or paclitaxel treatment, lysates from treated cells were immunoblotted with antibodies directed to active form of caspase 8 (Figure 4A) and caspase 3 (Figure 4B). As shown in Figure 3A and B, caspases 3 and 8 were not activated by ascites or paclitaxel treatment. In parallel, activities of caspase 3 and 7 were determined in SKOV3 cells treated or not with paclitaxel or ascite. As observed in Figure 4C, ascites treatment did not induce caspase 3 or 7 activities of cells. In contrast, combining results from Figure 3B and C, paclitaxel induced caspase 7 activities in SKOV3 cells.

### Malignant ascites-dependent apoptotic pathway is JNK dependent

**Fig. 5:**
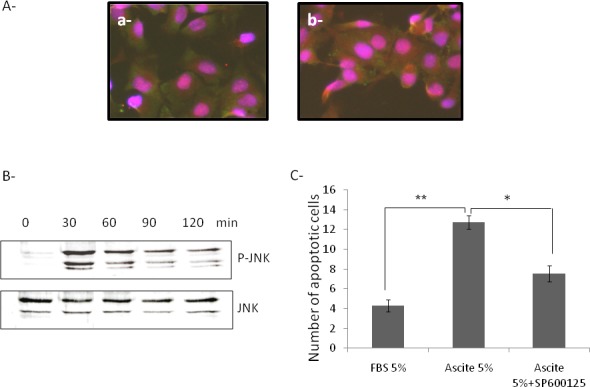
Involvement of JNK pathway in ascite-induced apoptosis (A) Evaluation of Daxx (red) and Fas (green) expression and localization in SKOV3 cells treated with 5% FBS (a) or 5% ascite (b) by immunofluorescence. Magnification X200 (B) Evaluation of phosphorylation kinetic of JNK in SKOV3 cells treated with 5% ascite by western blot analysis. (C) Evaluation of apoptotic cells in SKOV3 cells treated with 5% FBS (control), 5% ascite or 5% ascite+SP600125. *p≤0.05 **p≤0.01.

Besides the Caspase 8 signaling cascades, a number of other signaling pathways are also activated by Fas/ FasL interactions. For example, Death-Domain Associated protein (Daxx) binds to the Fas death domain and can enhance Fas-mediated apoptosis by activating the JNK kinase cascade [14]. The JNK kinase kinase Apoptosis Signal Regulating Kinase 1 (ASK1) sequesters Daxx in the cytoplasm and thus inhibits the repressive activity of Daxx in transcription [15]. In addition, Daxx was bound to the activated Fas only in the presence of ASK1, accelerating the Fas-mediated apoptosis. In the absence of ASK1, Daxx is found in the nucleus, where it localizes to Promyelocytic Leukemia Protein Oncogenic Domains. ASK1 and Daxx can induce a Caspase-independent form of cell death that is independent from the kinase activity of ASK1. In order to investigate the involvement of this signaling pathway, we treated cells with ascites and explored the localization of Daxx and kinetic of phosphorylation of JNK in these cells. As shown in Figure 5, ascites treatment induced the cytoplasmic expression of Daxx (Figure 5A) and phosphorylation of JNK within 30 minutes of treatment (Figure 5B). In parallel, we incubated cells with 5% ascites in presence or not of SP600125, an inhibitor of JNK pathway, to investigate the role of JNK activation in ascites-induced apoptosis. Ascites treatment of SKOV3 cells significantly induced the number of apoptotic cells compared to cell incubated with 5% FBS. The addition of JNK inhibitor partially reversed the effects of ascites on apoptosis of cells confirming the importance of JNK pathway in ascites-induced apoptosis (Figure 5C).

**Fig.6:**
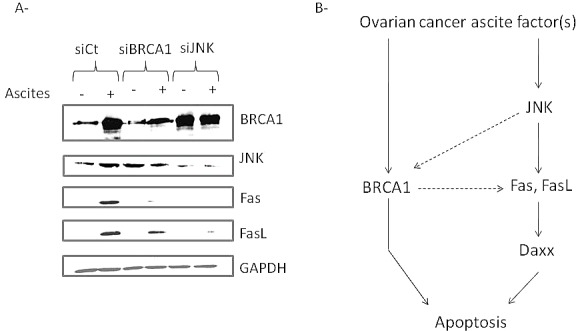
Ascites-induced JNK is placed upstream the induction of Fas and FasL expression (A) Evaluation of Fas and FasL expression in SKOV3 cells transfected with control, BRCA1 or JNK siRNA and incubated or not with 5% ascites. (B) Model of ascites-induced apoptosis in ovarian cancer cells. Malignant ascites could induce JNK pathway and thus, expression of BRCA1, Fas and FasL. BRCA1 may be also involved in Fas and FasL upregulation. Overexpression and cytoplasmic localization of Daxx allows sensitization of the Fas-mediated apoptosis. FasL binding to death receptor Fas results in induction of apoptosis.

To confirm the importance of JNK activation in ascites-induced apoptosis, we then down regulated expression of BRCA1 and JNK using siRNA strategy. As observed in Figure 6A, decreased expression of BRCA1 leads to a slight decrease in ascites-induced expression of Fas and FasL whereas decreased expression of JNK leads to a significant decrease in ascites-induced BRCA1, Fas and FasL expression suggesting that activation of JNK by ascites could be placed upstream BRCA1, Fas and FasL in the apoptotic pathway as hypothesized in Figure 6B.

### Investigation of oxidative stress involvement in ascites-induced apoptosis of SKOV3 cells

Because JNK is frequently activated by oxidative stress in cells [16], we investigated whether oxidative stress is involved in ascites-induced apoptosis of SKOV3 cells. We analyzed expression of 6 oxidative stress genes (NQO1, SOD1, EPHX1, MGST1, PRDX1 and GSR) in control and ascites-treated cells. None of these genes were induced by ascites treatment suggesting that malignant ascites do not induce oxidative stress in cells (supplementary data).

### Effects of EOC ascites on taxol-treatment of tumour

**Fig. 7:**
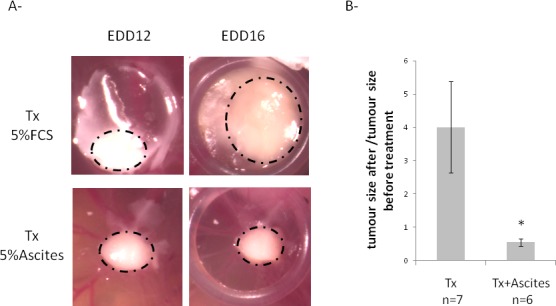
Effects of taxol and ascites on tumor development On EDD8, OCAM cells suspension were placed into the silicon O-ring for 4 days to allow tumor growth. On EDD12, tumors were treated with low dose of taxol in culture medium containing 5% FBS or 5% ascites for 4 days. Tumor size was monitored using a Wild Heerbrugg M3Z microscope at 10x magnification with a Lumenera INFINITY2-1 CDD camera with Infinity Capture Software. A- Representative photo of tumors obtained before (EDD12) and after treatment (EDD16). Dotted lines represent the demarcation of tumors. B- Evaluation of tumor growth after treatment.

Since EOC ascites could induce cell apoptosis and synergically act with taxol on cell viability in vitro, we hypothesized that ascites can be used in combination with taxol-treatment to increase its effect on tumor regression. To test this possibility, tumors were grown on the CAM and then treated at EDD12 for 4 days with low dose of taxol in combination or not with malignant ascites. At this taxol dose, tumors continued to grow (Figure 7A and B). In the opposite, when malignant ascites were added to taxol treatment, tumors size slightly regressed (Figure 7A and B).

## DISCUSSION

The present study supports that ascites from ovarian cancer patients regulate neither invasion nor proliferation of SKOV3 cells. This is in contrast with another detailed report which suggests that ovarian cancer ascites influence cell migration, proliferation and invasion. However, these effects are really different from one ascites sample to the other one [3, 7]. Difference of effects on migration and invasion of the ascites could be due at least in part to the fact that these authors did not normalize their results according to proliferation. Moreover, ascites from different types of ovarian cancer meaning different biological relevance, different grades and stages were compared. In the present study, we only focused on ascites from patients with high grade EOC.

We demonstrated for the first time that the acellular fraction of ascites enhances apoptosis of ovarian cancer cells, and acts in synergy with anticancer paclitaxel therapy to induce mortality both in vitro and in vivo. These effects are reproducible from one ascites to the other suggesting a general effect of malignant ascites on cell apoptosis.

The extrinsic apoptotic pathway is known to be induced by binding of death ligands from the tumor necrosis factor family to their specific cell surface receptors. FasL is a member of this TNF family [17] and binds to its agonistic specific receptor Fas. Alteration of the Fas/FasL system has been implied in carcinogenesis [18, 19] and malignant transformation of the ovaries [20]. More recently, it was shown that ovarian tumors that have retained Fas expression are better differentiated and have a better progression-free and disease-specific survival showing the importance of death receptor-targeted therapies in ovarian cancer [21]. Consequently, several approaches targeting the Fas/FasL signaling have been developed for cancer therapy [22]. Interestingly, here, we found that Fas and FasL levels are induced upon ascites treatment of ovarian cancer cells, suggesting thus a beneficial effect of the acellular fraction of ascites against ovarian cancer.

We then showed that the extrinsic pathway induced by ascites occurs through a JNK signaling pathway followed by an increase in BRCA1, Fas, FasL levels and an increased of cytoplasmic localization of Daxx. We also proved that this pathway is independent of caspase signaling pathway or oxidative stress. Previous studies had shown that BRCA1 could regulate both FasL and Fas expression in certain conditions [11, 23] and that JNK could be placed upstream of FasL and Fas in the apoptotic signaling pathway [11, 24, 25]. Using JNK siRNA transfection in SKOV3 cells, we confirmed that JNK is placed upstream of FasL and Fas in the apoptotic pathway and proposed a model of ascites-induced apoptosis via induction of JNK pathway and BRCA1 expression (Figure 6B).

In contrast to these observations, Lane et al. described ovarian malignant ascites as having a prosurvival activity by inhibiting TRAIL-induced apoptosis [4]. This could occurr through activation of the PI3K/Akt pathway in human ovarian carcinoma cells [6]. These observations are in contrast to ours and it is difficult to explain these discrepancies. One possible explanation is that effects of the malignant ascites observed by Lane et al. were really different from one to the other ascites, probably due to large heterogeneities of ascites origin (histopathology, grade and stage difference) [4, 5]. Moreover, most of the experiments focused only on ascites exhibiting an inhibition of TRAIL-induced apoptosis [4-6]. The importance of homogeneity of samples group is reinforced by the fact that contradictory results were obtained among their own studies. In their first study, authors observed that peritoneal fluid obtained from patients with benign gynecological disorders did not provide protection against TRAIL-induced apoptosis in CaOV3 cells in contrast to malignant ascites [6]. However, in their second study, more than half of benign ascites tested on CaOV3 cells increased TRAIL IC_50_ [5]. In addition, in their first study, they concluded that malignant ascites had no effect on cisplatin-mediated CaOV3 cell death whereas they described an increased of cisplatin IC_50_ by malignant ascites treatment of CaOV3 cells in their second study.

In summary, our work demonstrates that treatment with malignant ascites significantly enhances cell apoptosis and paclitaxel-induced mortality in ovarian cancer cells. This was also confirmed in vivo. In this context, acellular fractions of ascites could be beneficial to fight ovarian cancer, particularly for patients treated with paclitaxel.

Considering that malignant ascites also contain disseminating cancer cells, and is associated with unwanted symptoms, including abdominal pressure and distension, dyspnea, bloating, pelvic pain, and bowel/ bladder dysfunction which have to be managed in order to maintain overall quality of life of patients, cell-free and concentrated ascites re-infusion therapy (CART) could be envisaged for the treatment of patients with advanced ovarian cancer. This data corroborates a recent clinical study showed that CART can effectively improve the quality of life and survival of patients with advanced gynecological cancer [26].

## MATERIALS AND METHODS

### Ascites

The study was approved by the ethics committee and a written informed consent was obtained from patients. Ascites were obtained from 8 women with serous ovarian cancer (grade 2 and 3). They were centrifuged at 2400 rpm for 10 minutes and acellular fractions (supernatant) were collected, aliquoted and stored at −20°C.

### Cell culture

SKOV3 cells (EOC cell line) and OCAM cells (ovarian borderline tumor cell line) were grown in Mc Coy medium supplemented with 10% (v/v) fetal bovine serum (FBS), and gentamicin under 5% CO2, at 37°C. 24h after seeding, cells were cultured in McCoy medium supplemented with 5% FBS or 5% ascites for 48h.

To study paclitaxel induced-apoptosis, 100 nM paclitaxel were added in culture medium for 24h and 48h.

### Western Blot

Whole cell extracts (40 μg of proteins) were fractionated by SDS-Page 10% and transferred to nitrocellulose membrane for immunoblot analysis using rabbit anti-BRCA1 antibodies (1:1000, SantaCruz Biotechnology), mouse anti-Fas and anti-FasL antibodies (dilution 1:250 from SantaCruz Biotechnology), rabbit anti-caspase 3 antibodies (1:1000 from Cell Signalling), rabbit anti-caspase 8 antibodies (1:1000 dilution from Acris antibodies), rabbit anti-Phospho JNK or JNK antibodies (1:3000 dilution from Cell Signalling) and mouse anti-GAPDH antibodies (1:30 000 dilution from Millipore, Temecula, CA, USA).

### Invasion assay

Cell invasion assay was performed in an invasion chamber as described elsewhere [27]. Briefly, 3 × 10^4^ of SKOV3 cells in 100 μl of medium were added to the upper compartment of the transwell chambers. Medium (400 μl) was added in the lower chamber for 12h at 37°C in a CO_2_ (5%) incubator. Cells were incubated with 5% FBS (control) or 5% ascites for 48 hours. After incubation, viable cells that invaded collagen were stained with crystal violet and measurement was performed at 560 nm. This assay was repeated three times and each experiment was run in triplicate with two different antibodies purification. Data were expressed as the percentage of treated cells that invaded the collagen-coated membrane compared to the untreated (controls) cells.

### Proliferation assay

Cell proliferation was assessed by Wst-1 assay following the manufacturer's instructions (Roche) and performed at the same time and with the same preparation of cells as the one used for the invasion assay. Briefly, 3 × 10^4^ SKOV3 cells were seeded in 96-well plates and incubated in 100 μl of medium for 24h at 37°C in a CO_2_ (5%) incubator. The incubation culture medium was then removed and replaced by 100 μl of medium containing 5% FBS (as control) or 5% ascites for 48 hours before being replaced with medium containing 10% Wst-1 reagent for 30 minutes. Microplate was then read at 450 nm.

### Viability assay

Cell viability was determined by MTT assay. SKOV3 (3 × 10^4^ cells) or OCAM (1.5 × 10^4^) cells were seeded in 96-well plates and incubated in 100 μl of medium for 24h at 37°C in a CO_2_ (5%) incubator. The incubation culture medium was then removed and replaced by 100 μl of medium containing 5% FBS (as control) or 5% ascites in presence or not of 100 nM paclitaxel for 24 or 48 hours before being replaced with medium containing 20% MTT (Sigma-Aldrich Corporation, USA) solution (5mg/ml in medium) for 3h. Acidic isopropanol solution (150 μl) was added, and then each well was vigorously mixed to dissolve the precipitated formazan. UV–visible absorption was measured at 560 and 690 nm.

### Early apoptosis assay

Apoptosis was measured with the apoptosis assay APOPercentage from Biocolor as previously described [27]. Briefly, 3 × 10^4^ SKOV3 cells were seeded in gelatine-coated 96 wells and incubated in 100 μl of medium at 37°C, 5% CO2 until confluence was reached (24h). The incubation culture medium was then removed and replaced by 100 μl of fresh medium containing 5% FBS (as control) or 5% ascite in presence or not of H_2_O_2_ for 6 hours before addition of 5 μl of APOPercentage Dye for 30 minutes at 37°C and 5% CO2. Culture medium was then removed, and cells were gently washed with PBS to remove unbound dye. Then, either red cells were counted under microscopy or 100 μl of Dye releasing reagent was added to the wells to extract bound dye. Wells were shaken for 20 minutes before being read at 540 nm.

### Caspase 3/7 activities

Caspase 3/7 activity was measured with Caspase-Glo assay kit (Promega, Dubendorf, Switzerland). SKOV3 cells (3 × 10^4^ cells) were seeded in 96-well plates and incubated in 100 μl of medium for 24h at 37°C in a CO_2_ (5%) incubator. The incubation culture medium was then removed and replaced by 50 μl of medium containing 5% FBS (as control) or 5% ascites in presence or not of 100 nM paclitaxel for 48 hours. Cells were removed from the incubator and allowed to equilibrate at room temperature for 30 min. A volume of 50 μl of Caspase-Glo reagent was added to each well. The plate was then incubated at room temperature for 1 h. Luminescence of each sample was measured in a plate-reading luminometer (Glomax, Promega, Dubendorf, Switzerland) with parameters of 1-min lag time and 0.5 s/well reading time. The experiments were performed three times in triplicate.

### Immunofluorescence

SKOV3 cells were plated in labtek chambers and treated 24h later with 5% FBS or 5% ascites for 48h. Cells were washed with PBS, fixed with 3% paraformaldehyde for 10 minutes, washed again with PBS and permeabilized with Triton 0.5% in PBS for 10 minutes. Non-specific binding was blocked with 5% v/v BSA in PBS for 30 min at room temperature. They were then incubated with the anti-fas and anti-Daxx antibodies (SantaCruz, Labforce, Switzerland) or with IgG isotypic control overnight at 4°C. The slides were washed four times with TBS and incubated with the appropriate fluorescent secondary antibody: anti-mouse Alexa Fluor 488 or anti-rabbit Alexa Fluor 568 (1 h, room temperature). DAPI was used to stain the nucleus of cells. Images were visualized with a microscope (Axiocam Fluo).

### siRNA transfection

To determine the role of BRCA1 and JNK on ascites-induced Fas and FasL expression, 1 million of cells were plated in 60 mm dishes and then transfected with BRCA1 (SantaCruz Biotechnology), JNK (Cell Signalling, Bioconcept, Allschwil, Switzerland) or control siRNA (SantaCruz Biotechnology) using the Interferin tranfection reagent (Polyplus transfection Illkrich, France) and following manufacturer's protocol. After 24h, cells plated in 60 mm dishes were trypsinized and seeded in 3 cm dishes in culture medium containing 5% FBS or 5% ascites and incubated for 48h.

### Tumor development and treatment on chick chorioallantoid membrane (CAM) Chick embryo culture

Fertilized eggs (animal facility of the University of Geneva, Geneva, Switzerland) were incubated at 38°C with 80% relative humidity and periodic rotation. Rotation was stopped on egg development day (EDD) 4 and eggs were drilled at their narrow apex. The hole was closed with adhesive tape. Incubation was carried out until use.

### Cells grafting

On EDD8 the hole in the eggshell was enlarged to allow the access to the CAM. After gently scratching of the membrane with a needle tip, a silicon O-ring (Apple Rubber products inc., Lancaster, USA) was placed onto a blood vessel crossing. OCAM cells suspension (2.5X10^6^ cells in 30 ul) were placed into the silicon O-ring and the hole was hermetically covered with Parafilm®. Eggs were returned to the incubator for 4 days to allow tumor growth. Tumor growth was monitored using a Wild Heerbrugg M3Z microscope at 10x magnification with a Lumenera INFINITY2-1 CDD camera with Infinity Capture Software.

### S2 Treatment

Four days after the grafting (EDD12), available tumors were treated topically with 2.5 μM taxol diluted in culture medium containing 5% FBS or 5% ascites. Eggs were returned to the incubator for 4 days (EDD16). Tumor size was monitored using a Wild Heerbrugg M3Z microscope at 10x magnification with a Lumenera INFINITY2-1 CDD camera with Infinity Capture Software.

### Statistical analysis

Results were expressed as mean +/− standard deviation. The difference between samples was evaluated by the student t test. A p<0.05 was considered as statistically significant.

## SUPPLEMENTARY MATERIALS FIGURE


