# Supplementary material for: Acellular fraction of ovarian cancer ascites induce apoptosis by activating JNK and inducing BRCA1, Fas and FasL expression in ovarian cancer cells
Source: Oncoscience. 2014 Apr 30;1(4):262–71. doi: 10.18632/oncoscience.31 (PMC4278302; doi:10.18632/oncoscience.31)
Supplement: Supplementary file 1 [file oncoscience-01-0262-s001.docx]

**Supplementary Materials**

Materials and Methods

*qRT-PCR*

Reverse transcription was performed with 1 µg of total RNA in a final volume of 20 µl using High Capacity cDNA Reverse Transcription Kit (Applied biosystems, Foster city, USA). The quantitative detection of the PCR product was performed using the KAPA SYBR FAST Universal qPCR Kit (KAPA Biosystems, Boston, USA), with the iCycler iQ System (Bio-Rad).

The relative expression was normalized to the housekeeping gene GAPDH.

Oligonucleotide primers for qPCR were as follows: human GAPDH forward 5'-CAA GGG CAT CCT GGG CTA-3' and reverse 5'-TTG AAG TCA GAG ACC ACC TG-3', human NQO1 forward 5'-TGA AGG ACC CTG CGA ACT TTC-3' and reverse 5'-GAA CAC TCG CTC AAA CCA GC-3', human PRDX1 forward 5'-GGG ACC CAT GAA CAT TCC TTT-3' and reverse 5'-TGA TCT GCC GAA GAA TAC C-3', human GSR forward 5'-AGT GAT CCC AAG CCC ACA ATA-3' and reverse 5'-CAG CAA TGT AAC CTG CAC CAA-3', human SOD1 forward 5'-AGG GCA TCA TCA ATT TCG AGC-3' and reverse 5'-GCC CAC CGT GTT TTC TGG A-3', human EPHX1 forward 5'-GTC ATC TCC TAC TGG CGG AA-3' and reverse 5'-CTT CAC GTG GAT GAA GTG GA-3', human MGST1 forward 5'-ATG ACA GAG TAG AAC GTG TAC GC-3' and reverse 5'-ATG GCT GTA GAG GGG TCG G -3'.

**Fig. S1.** **Stress oxidative mRNA level genes evaluated by qRT-PCR.**

Evaluation of 6 different oxidative stress genes mRNA in SKOV3 cells treated with 5% FBS (control, CT) or 5% ascites for 48h by RT-qPCR in SKOV3 cells.
